# Effects of FK506 on Ca^2+^ Release Channels (Review)

**DOI:** 10.4137/pmc.s382

**Published:** 2008-03-18

**Authors:** Terutaka Ozawa

**Affiliations:** Department of Physiology, Tohoku University Graduate School of Medicine, 2-1 Seiryo-machi, Aoba-ku, Sendai 980-8575, Japan

**Keywords:** ryanodine receptor, IP_3_ receptor, FK506, FKBP

## Abstract

Tacrolimus (FK506), which was isolated from the fermentation broth of Streptomyces tsukubaensis No. 9993, has an immunosuppressive effect. In T-lymphocytes, FK506 binds to the intracellular receptor, a 12-kDa FK506-binding protein (FKBP12). The FK506-FKBP12 complex binds to the phosphatase calcineurin (CN) and inhibits the activity of CN. By inhibition of the activity of CN, dephosphorylation of a nuclear factor of activated T-cells (NFAT) is inhibited, and translocation of the NFAT to the nucleus is suppressed. Thereby, the production of T-cell-derived mediators such as interleukin 2 (IL-2) is inhibited, and the proliferation of cytotoxic T-cells is suppressed. In muscle cells, FKBP12 and FKBP12.6 are associated with ryanodine-sensitive Ca^2+^ release channels (ryanodine receptors: RyRs) on the skeletal and cardiac muscle sarcoplasmic reticulum (SR), respectively. FK506 modulates the RyR by dissociating FKBP12 or FKBP12.6 from the RyR complex. FKBP12 is also associated with inositol 1,4,5-trisphosphate (IP_3_)-sensitive Ca^2+^ release channels (IP_3_ receptors: IP_3_Rs) on the endoplasmic reticulum (ER) of non-muscle cells. The IP_3_R-FKBP12 complex binds to CN, which dephosphorylates the protein kinase C (PKC) phosphorylation site on the receptor. When FKBP12 is dissociated from the IP_3_R complex by FK506, CN is also dissociated from the IP_3_R. Thereby, the IP_3_R is phosphorylated by PKC, and the receptor is modulated. Recently, it was found that FK506 itself induces Ca^2+^ release through RyRs in some tissues.

## Introduction

The immunosuppressant tacrolimus (FK506) was isolated from the metabolite of a streptomycete in 1984 as a drug to inhibit T-cell activation [1]. In T-lymphocytes, FK506 binds to the intracellular receptor, a 12-kDa FK506-binding protein (FKBP12) [2, 3]. The FK506-FKBP12 complex binds to the Ca^2+^-activated phosphatase calcineurin (CN) and inhibits the activity of CN [4, 5]. CN dephosphorylates a nuclear factor of activated T-cells (NFAT) in the cytoplasm [6]. The dephosphorylated NFAT is translocated to the nucleus [7] and induces the production of T-cell-derived mediators such as interleukin 2 (IL-2) via transcription of the gene. FK506 prevents the translocation of an NFAT to the nucleus via the inhibition of CN activity [7]. Therefore, the production of IL-2, which induces the proliferation of cytotoxic T-cells, is inhibited.

It was shown in the 1990’s that FKBP12 is tightly associated with ryanodine-sensitive Ca^2+^ channels (ryanodine receptors: RyRs) on the sarcoplasmic reticulum (SR) of skeletal muscle [8, 9]. FK506 has been shown to promote dissociation of FKBP12 from the RyR complex [10] and to increase Ca^2+^ release through the channel [10, 11]. In the cardiac muscle SR, FKBP12.6 is associated with RyRs [12, 13]. FKBP12 has been shown to be associated also with inositol 1,4,5-trisphosphate (IP_3_)-sensitive Ca^2+^ channels (IP_3_ receptors: IP_3_Rs) on the endoplasmic reticulum (ER) of cerebellum tissues [14, 15]. The IP_3_R-FKBP12 complex binds to CN [15, 16], which dephosphorylates the protein kinase C (PKC) phosphorylation site on the IP_3_R [15]. When FKBP12 is dissociated from the IP_3_R complex by FK506, CN is also dissociated from the IP_3_R [15]. Thereby, the IP_3_R is phosphorylated by PKC, and Ca^2+^ release through the receptor is increased [14]. Recently, it was shown that FK506 itself induces Ca^2+^ release from the RyR in pancreatic acinar cells, probably via an FKBP-independent mechanism [17, 18].

In this review, the effects of FK506 as an immunosuppressant are described briefly, and the effects of FK506 on Ca^2+^ release channels of the SR or ER membrane are described in detail.

## Chemicals

**Figure 1:**
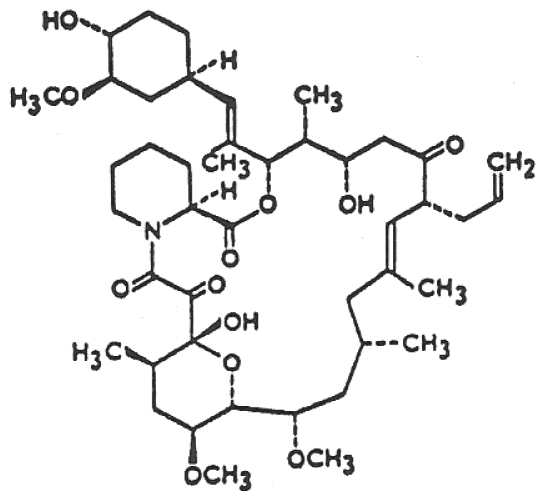
Chemical structure of tacrolimus (FK506).

Tacrolimus (Fig. 1), a 23-membered macrolide lactone, was isolated from the fermentation broth of Streptomyces tsukubaensis No. 9993 in 1984 [1] and is designated by the code number of FK506. The drug is a colorless prism, and the molecular formula is C_44_H_69_NO_12_·H_2_O (Mw: 804.02). The chemical structure was determined by Tanaka et al. [19]. The drug is soluble in ethanol, methanol and DMSO and is insoluble in water.

## Effects of FK506 as an Immunosuppressant

Cyclosporin A (CsA), a fungal metabolite, was a powerful immunosuppressive agent in the 1980’s and has been used as a therapeutic agent to prevent graft rejection following organ transplantation. CsA inhibits the production of T-cell-derived soluble mediators such as IL-2, which is induced by antigen stimulation in T-lymphocytes. FK506 inhibits the production of IL-2 at about a one hundred-times lower concentration than that of CsA [20]. CsA and FK506 bind to intracellular receptors named cyclophilins [21, 22] and FKBPs [2, 3], respectively. Both receptors, called immunophilins, are abundant, ubiquitous proteins within cells and comprise a family of proteins [23]. All immunophilins have *cis-trans* peptidyl-prolyl isomerase (PPIase) activity. It has been shown that the PPIase activity is inhibited by the binding of CsA and FK506 to immunophilins [2, 3, 24, 25]. However, the PPIase activities of cyclophilins and FKBPs are known to be unrelated to the immunosuppressive activities of CsA and FK506, respectively. The major proteins of cyclophilins and FKBPs are cyclophilin A (CypA) and FKBP12, respectively. Both the CsA-CypA and FK506-FKBP12 complexes can bind to the Ca^2+^-activated phosphatase CN and inhibit the enzyme activity of CN [4, 5]. CN is activated by the increment of intracellular Ca^2+^ concentrations following T-cell receptor stimulation by antigens and dephosphorylates a cytosolic component of NFAT (NFAT_c_) in the cytoplasm [6]. The NFAT_c_ dephosphorylated by CN is translocated to the nucleus [7] and combines with a nuclear component of NFAT (NFAT_n_) [7, 26]. The NFAT_c_-NFAT_n_ complex binds to the regulatory site on the IL-2 promoter to activate transcription of the IL-2 gene [27]. CsA and FK506 inhibit the translocation of NFAT_c_ to the nucleus via inhibition of CN activity [7] and therefore prevent transcription of the IL-2 gene. As a result of the inhibition of IL-2 production, the proliferation of cytotoxic T-cells that contribute to the destruction of target tissues is suppressed.

## Ca^2^^+^ Release Channels

In a variety of cell types, intracellular Ca^2+^ stores play an essential role in the regulation of cytosolic Ca^2+^ concentration, the elevation of which triggers many cellular events, such as muscle contraction, enzyme secretion, cell proliferation and egg fertilization. Two distinct classes of Ca^2+^ release channels, which induce the release of Ca^2+^ from the stores into the cytosol, have been identified.

One is sensitive to the ubiquitous second messenger IP_3_ that is formed by stimulation of a cell surface receptor with hormones or neurotransmitters [28]. Ca^2+^ channels (receptors) sensitive to IP_3_ (IP_3_Rs) are widely distributed on the ER of many tissues, including T-lymphocytes. The channel protein has been purified [29] and cloned [30, 31] in brain tissues. Three IP_3_R isoforms (IP_3_R1, IP_3_R2 and IP_3_R3) are expressed [32, 33]. IP_3_R1, the major type of IP_3_R, is widely expressed in the rodent brain, predominantly in cerebellar Purkinje cells. IP_3_R2 is expressed in glial cells. IP_3_R3 is expressed in the kidney, pancreatic islets and intestinal epithelium.

The other is sensitive to the plant alkaloid ryanodine. Ca^2+^ channels (receptors) sensitive to ryanodine (RyRs) are activated by caffeine, ryanodine and Ca^2+^. The channel protein has been purified [34, 35] and cloned [36, 37] in the skeletal and cardiac muscle SR. RyRs were also characterized in the ER of non-muscle cells, including brain cells [38], liver cells [39] and exocrine cells [40]. It has been shown that three RyR isoforms (RyR1, RyR2 and RyR3) are expressed [41–43]. RyR1 and RyR2 have been found to be localized in skeletal muscle and cardiac muscle, respectively, while RyR3 is found in the brain and smooth muscle. Recently, it was shown that the endogenous NAD^+^ metabolite cyclic ADP-ribose (cADPR) induces Ca^2+^ release from RyRs in sea urchin eggs [44, 45], cardiac muscle cells [46], brain cells [47] and pancreatic β cells [48]. This compound is thought to be an intracellular messenger in addition to IP_3_ [49]. It has also been shown that a low concentration of cADPR can modulate the RyR [17, 50, 51].

## Effects of FK506 on Ca^2^^+^ Release Channels

FK506 is known to modulate RyRs. In the skeletal muscle SR, RyR1 is tightly associated with FKBP12 [8, 9]. It has been found that one mole of FKBP12 is associated with each protomer of homotetrameric RyR1 [10]. In association with RyR1, FKBP12 has been shown to stabilize the closed conformation of the Ca^2+^ release channel [10]. FK506 has been shown to promote dissociation of FKBP12 from the RyR1 complex [10]. The EC_50_ value for dissociation of FKBP12 from the RyR1 complex in skeletal muscle has been reported to be in the concentration range of 0.12 to 0.5 μM FK506 [10]. By the removal of FKBP12, RyR1 exhibits subconductance states [11], and the Ca^2+^ or caffeine sensitivity of the channel is enhanced [10, 52]. Compared with control SR vesicles, FKBP12-deficient SR vesicles have been shown to increase open probability and mean open times for single channel recordings of the receptor [52].

In cardiac type RyR (RyR2), FKBP12.6 binds to the receptor [12, 13, 53]. FK506 is known to activate or modulate RyR2 by the removal of FKBP12.6. FKBP12.6-deficient cardiac SR Ca^2+^ channels did not increase the open probability for single channel recordings as was the case of FKBP12-deficient skeletal SR Ca^2+^ channels [13]. Although the type of RyR is unclear, it has been shown that FK506 (0.1–100 μM) increases the open probability of reconstituted RyRs (Ca^2+^ channels) in coronary arterial smooth muscle cells, in which FKBP12.6 was detected [54]. This result suggests that FK506 activates the RyR in this tissue by the removal of FKBP12.6. It has been shown that FK506 at a micromolar concentration range induces Ca^2+^ release from RyR2 of pancreatic islet microsomes by dissociating FKBP12.6 [53]. Recently, it was shown that FK506 (3 μM) shifts the dose-response curve of ryanodine- or caffeine-induced ^45^Ca^2+^ release from the microsomal vesicles of rat pancreatic acinar cells to the left [17]. Since an RyR2 isoform has been identified in rat pancreatic acinar cells [55, 56], FKBP12.6 may be involved in the modulation of Ca^2+^ release through the RyR by FK506. It has been found that cADPR as well as FK506 can bind to FKBP12.6 and dissociate FKBP12.6 from pancreatic islet microsomes to release Ca^2+^ [53]. An antibody against FKBP12.6 has been shown to inhibit activation of the RyR induced not only by FK506 but also by cADPR in coronary arterial smooth muscle cells [54]. These findings suggest that cADPR dissociates FKBP12.6 from the RyR-FKBP12.6 complex to activate the Ca^2+^ channel. It has been found in rat pancreatic acinar cells that cADPR shifts the dose-response curve of ryanodine- or caffeine-induced ^45^Ca^2+^ release to the left by the same extent as that in the case of FK506 and that the stimulatory effects on ryanodine- or caffeine-induced ^45^Ca^2+^ release by cADPR and by FK506 are not additive [17]. The results suggest that cADPR modulates the RyR in pancreatic acinar cells by the same mechanism as that by which FK506 modulates the RyR. The endogenous ligand cADPR might induce activation or modulation of the RyR by the removal of FKBP12.6 from the RyR complex under physiological conditions. It has been shown that protein kinase A (PKA), which is activated by adrenergic stimulation of cardiac muscle cells, phosphorylates Ser 2809 on RyR2 of the canine heart [57]. The RyR2 phosphorylated by PKA dissociates FKBP12.6 from the receptor and increases open probability of the channel [57, 58]. In heart failure, the adrenergic receptor is chronically stimulated. The phosphorylation of RyR2 by PKA in failing hearts of humans and canines is increased by ∼4 fold compared with that in nonfailing hearts [57]. The hyperphosphorylation of RyR2 by PKA in failing hearts results in a depletion of FKBP12.6 from the RyR2 complex [57, 59] and an abnormal Ca^2+^ leak through the RyR2 [60, 61].

IP_3_R1, a structurally related tetramer that has up to 40% sequence identity with RyR1 [30, 31, 33], associates with FKBP12 [14, 15]. It has been found that FKBP12 binds to the IP_3_R1 of the rat cerebellum at residues 1400–1401, a leucyl-prolyl dipeptide that is an FK506-like domain [16]. The EC_50_ value for dissociation of FKBP12 from the IP_3_R1 complex in the rat cerebellum has been shown to be in the concentration range of 10 to 100 nM FK506 [15]. The IP_3_R1-FKBP12 complex can associate with CN [15, 16]. CN has been shown to dephosphorylate the PKC phosphorylation site on IP_3_R1 [15]. When FKBP12 is dissociated from the IP_3_R1 complex by FK506, binding of CN to the FK506-FKBP12 complex is stimulated [15]. Thereby, IP_3_R1 is phosphorylated by PKC, and Ca^2+^ flux through IP_3_R1 is increased [14]. Under physiological conditions, increase in Ca^2+^ release from the ER to the cytosol through the IP_3_R by PKC activation activates CN, and the activated CN decreases Ca^2+^ release by dephosphorylation of the receptor. Thus, the increase and decrease in Ca^2+^ release through IP_3_R mediate Ca^2+^ oscillations [15].

It is known that FK506 itself induces Ca^2+^ release from the ER of non-muscle cells. It has been shown that FK506 induces Ca^2+^ release through the RyR in pancreatic islets by the removal of FKBP12.6 as mentioned above [53]. Recently, it was shown that FK506 induces a biphasic ^45^Ca^2+^ release from the ER of pancreatic acinar cells [18]. The first phase of the FK506-induced ^45^Ca^2+^ release was seen at concentrations up to 10 μM (K_m_ = 0.5 μM), and the second phase of the release was seen at concentrations over 10 μM (K_m_ = 55 μM). The first phase of the release was stimulated by the presence of cADPR [17]. The FK506-induced response caused by the dissociation of FKBP should be reduced in the presence of cADPR, since it is thought that FKBP had been removed from the RyR by cADPR before the addition of FK506. It is unlikely that the FK506 (≤ 10 μM)-induced ^45^Ca^2+^ release in pancreatic acinar cells is due to the dissociation of FKBP. FK506 is a compound with a macrocyclic lactone ring structure (Fig. 1). It has been shown that rapamycin and ivermectin, macrocyclic lactone derivatives, increased the open probability of FKBP12-stripped RyRs in skeletal muscle [62, 63]. This finding suggests that the compounds activate the RyR by a mechanism other than dissociation of FKBP. The first phase of the FK506-induced ^45^Ca^2+^ release in pancreatic acinar cells may be explained by a direct activation of the RyR by FK506. The second phase of the FK506-induced ^45^Ca^2+^ release in pancreatic acinar cells was inhibited by heparin, an inhibitor of the IP_3_R [18]. Although it is unclear whether FKBP is involved in the second phase of the FK506-induced release, there is a possibility that FK506 (>10 μM) directly activates the IP_3_R in pancreatic acinar cells.

## Conclusion

FKBPs are abundant and ubiquitous proteins within cells. FKBP12 or FKBP12.6 binds to the RyR on the SR or ER and regulates Ca^2+^ release from the receptor to the cytosol. FK506 dissociates FKBP12 or FKBP12.6 from the RyR complex and increases Ca^2+^ release through the receptor. In addition, the FK506-FKBP12 complex binds to the phosphatase CN and inhibits the enzyme activity of CN. In T-lymphocytes, dephosphorylation of an NFAT by CN is inhibited by FK506. Therefore, translocation of the NFAT to the nucleus is inhibited, and the immunoreaction of T-cells is suppressed. FKBP12 and CN bind to the IP_3_R on the ER. When FKBP12 is dissociated from the IP_3_R complex by FK506, CN is also dissociated from the receptor. Thereby, the IP_3_R is phosphorylated by PKC, and the Ca^2+^ release through the IP_3_R is increased. FK506 has been used as a tool to elucidate the pathway of immunoreaction and the modulation mechanism of Ca^2+^ release from the SR or ER. With elucidation of the action of FK506 and the cellular functions of FKBPs, FK506 may become more widely used in biomedical research.

## Abbreviations

cADPR: cyclic ADP-ribose
CN: calcineurin
CsA: cyclosporin A
CypA: cyclophilin A
ER: endoplasmic reticulum
FKBP: FK506-binding protein
IL-2: interleukin 2
IP_3_: inositol 1,4,5-trisphosphate
IP_3_R: IP_3_ receptor
NFAT: nuclear factor of activated T-cells
PKA: protein kinase A
PKC: protein kinase C
PPIase: *cis-trans* peptidyl-prolyl isomerase
RyR: ryanodine receptor
SR: sarcoplasmic reticulum
